# Effectiveness of ozone generated by a dielectric barrier discharge plasma reactor against multidrug-resistant pathogens and *Clostridioides difficile* spores

**DOI:** 10.1038/s41598-022-18428-w

**Published:** 2022-08-18

**Authors:** Cheolwoo Bong, Ji Young Choi, Jinseung Bae, Sungsu Park, Kwan Soo Ko, Moon Soo Bak, Hae Suk Cheong

**Affiliations:** 1 Sungkyunkwan University School of Mechanical Engineering, 2066, Serbu-ro, Jangan-gu, Suwon-si, Gyeonggi-do Republic of Korea; 2 Biomedical Institute for Convergence at SKKU (BICS), Sungkyunkwan University, Suwon, Korea; 3 Department of Microbiology, Sungkyunkwan University School of Medicine, Suwon, South Korea; 4 Division of Infectious Diseases, Department of Internal Medicine, Kangbuk Samsung Hospital, Sungkyunkwan University School of Medicine, 29, Saemoonan-ro, Jongro-gu, Seoul, 03181 Republic of Korea

**Keywords:** Microbiology, Bacteriology, Microbiology techniques

## Abstract

The contaminated healthcare environment plays an important role in the spread of multidrug-resistant organisms (MDROs) and *Clostridioides difficile.* This study aimed to evaluate the antimicrobial effects of ozone generated by a dielectric barrier discharge (DBD) plasma reactor on various materials that were contaminated by vancomycin-resistant *Enterococcus faecium* (VRE), carbapenem-resistant *Klebsiella pneumoniae* (CRE), carbapenem-*resistant Pseudomonas aeruginosa* (CRPA), carbapenem-resistant *Acinetobacter baumannii* (CRAB) and *C. difficile* spores. Various materials contaminated by VRE, CRE, CRPA, CRAB and *C. difficile* spores were treated with different ozone concentrations and exposure times. Atomic force microscopy (AFM) demonstrated bacterial surface modifications following ozone treatment. When an ozone dosage of 500 ppm for 15 min was applied to VRE and CRAB, about 2 or more log_10_ reduction was observed in stainless steel, fabric and wood, and a 1–2 log_10_ reduction in glass and plastic. Spores of *C. difficile* were more resistant to ozone than were all other tested organisms. On AFM, the bacterial cells, following ozone treatment, were swollen and distorted. The ozone generated by the DBD plasma reactor provided a simple and valuable decontamination tool for the MDROs and *C. difficile* spores, which are known as common pathogens in healthcare-associated infections.

## Introduction

The emergence of multidrug-resistant organisms (MDROs) is due to the misuse of antibiotics in humans and animals and it is defined as a serious threat to public health by the World Health Organization (WHO)^1^. Particularly, healthcare facilities are increasingly faced with the emergence and spread of MDROs. The main MDROs are methicillin-resistant *Staphylococcus aureus* and vancomycin-resistant enterococci (VRE), extended-spectrum beta-lactamase (ESBL)-producing *Enterobacterales*, multidrug-resistant *Pseudomonas aeruginosa*, multidrug-resistant *Acinetobacter baumanni* and carbapenem-resistant *Enterobacterales* (CRE). Also, *Clostridioides difficile* infection is the predominant cause of health care-associated diarrhoea and it places a significant burden on the healthcare system^2^. MDROs and *C. difficile* are transmitted through the hands of healthcare workers, the contaminated environment or directly from person to person. In recent studies, the contaminated healthcare environment has been shown to play an important role in the spread of MDROs and *C. difficile* when healthcare workers (HCWs) contaminate their hands by touching contaminated surfaces, or when patients come into direct contact with contaminated surfaces^3,4^. Therefore, cleaning the contaminated environments of healthcare facilities leads to a decreased rate of infection or colonisation by MDROs and *C. difficile*^5–7^. Given the worldwide concern about increasing antimicrobial resistance, it is clear that more studies on decontamination techniques and procedures for healthcare facilities are needed. Recently, no-touch methods for terminal cleaning, especially ultraviolet (UV) devices or hydrogen peroxide systems, have been regarded as promising decontamination methods. However, these commercially available devices using UV or hydrogen peroxide are not only expensive, but UV sterilisation has only been effective for exposed surfaces and plasma sterilisation with hydrogen peroxide has required a relatively long purging time until the next sterilisation cycle^5^.

Ozone has known antiseptic properties and can be produced inexpensively^8^. It is also known as toxic to human health, but can quickly be dissociated into oxygen^8^. Plasma reactors with dielectric barrier discharges (DBDs) are the most common ozone generating devices currently available^9^. DBD devices allow the generation of low temperature plasma in the air with the production of ozone. The practical application of ozone has been limited so far mainly to the disinfection of swimming pool water, drinking water and wastewater^10^. Few studies have reported its use in healthcare settings^8,11^.

In this study, we use a compact DBD plasma ozone generator to prove its effectiveness for decontaminating MDROs and *C. difficile*, and even those inoculated on various materials that are commonly used in healthcare settings. In addition, the sterilisation process with ozone was elucidated via atomic force microscopy (AFM) imaging of ozone-treated cells.

## Methods

### Bacterial strains and preparation of material coupons

The bacterial strains were prepared from clinical isolates: VRE (SCH 479 and SCH 637), carbapenem-resistant *Klebsiella pneumoniae* (CRE; SCH CRE-14 and DKA-1), carbapenem-resistant *Pseudomonas aeruginosa* (CRPA; 54 and 83) and carbapenem-resistant *Acinetobacter baumannii* (CRAB; F2487 and SCH-511). *Clostridioides difficile* was obtained from the National Culture Collection for Pathogens (NCCP 11840) in Korea Disease Control and Prevention Agency. It had been isolated in a patient from South Korea in 2019, and belongs to ST15 as determined in multilocus sequence typing. Brain–heart infusion (BHI) broth (BD, Sparks, MD, USA) inoculated with VRE, CRE, CRPA and CRAB was mixed thoroughly and incubated at 37 °C for 24 h.

*C. difficile* was streaked on blood agar anaerobically for 48 h. Several colonies were then added to 5 mL of brain–heart infusion broth and incubated anaerobically for 48 h. Thereafter, the culture was vortexed, missed with 5 mL of 95% ethanol, vortexed again and left at room temperature for 30 min. After centrifugation at 3000 × g for 20 min, the supernatant was discarded and the pellet containing spores plus killed bacteria was suspended in 0.3 mL of water. Viable cells were counted performed by spirally plating the bacterial cell suspension on blood agar plates after appropriate dilutions. Gram staining verified that 85% to 90% of the bacterial structures were spores^12^.

The following study was performed to investigate the effect of ozone as a disinfectant for various surfaces seeded with MDROs and *C. difficile* spores that are known to cause healthcare-associated infections. Stainless steel, fabric (cotton), glass, plastic (acrylic) and wood (pine tree) coupons, which were sized one centimetre by one centimetre were prepared. The coupons were sterilised before use. All coupons were disinfected by autoclaving before being contaminated by bacteria.

### Ozone sterilisation equipment with DBDs

**Figure 1:**
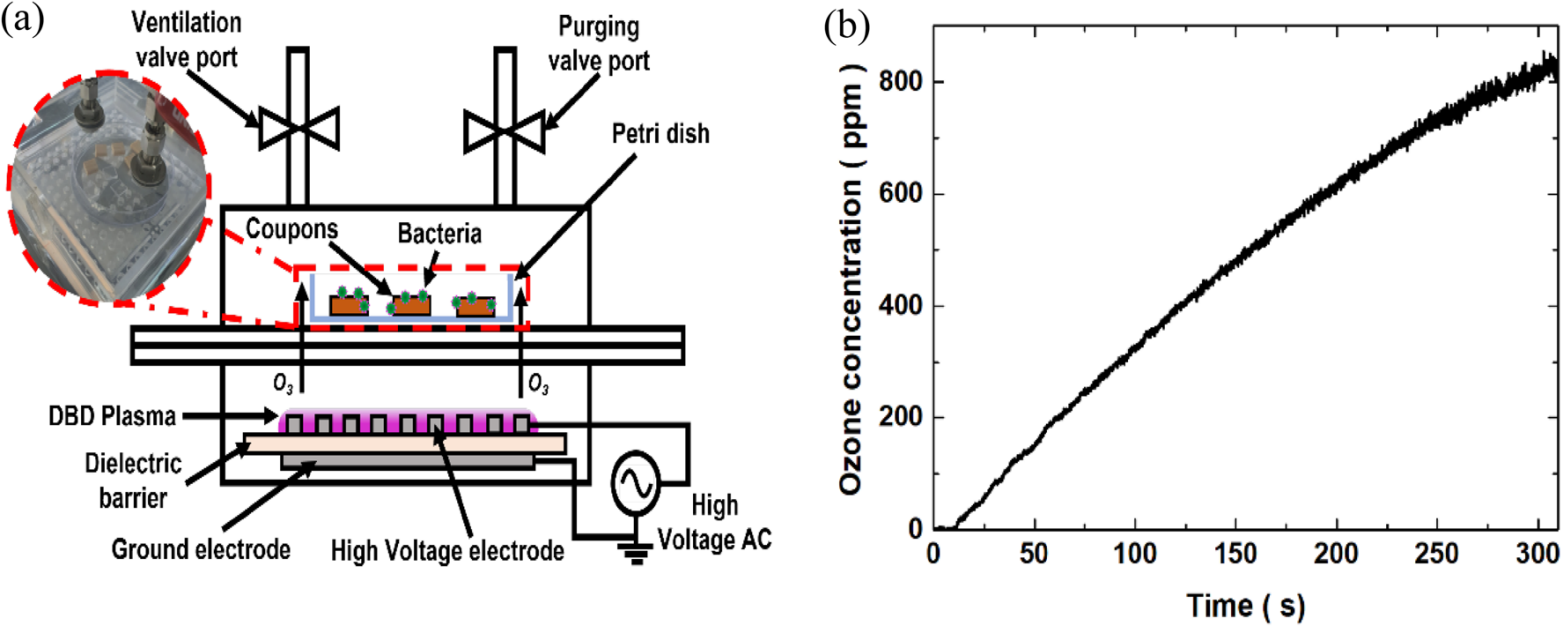
(**a**) Schematic of an experimental setup for sterilizing bacteria on various materials using ozone produced from a DBD plasma reactor, and (**b**) ozone concentration in the sterilization chamber with plasma generation time. The graph was plotted using the OriginPro version 9.0 (OriginPro software, Northampton, MA, USA; https://www.originlab.com).

In this study, bacterial cells were dispensed onto different substrate surfaces as well as agar plates. The plates were then sterilised by exposing them to ozone for a certain period and at a certain concentration in a sealed chamber. Figure 1 shows a photograph of the ozone sterilisation equipment. A plasma reactor with DBDs is made by attaching perforated and bare stainless steel electrodes in the front and back of a 1 mm thick alumina (dielectric) plate. For the perforated electrode, the pore size and open area were 3 mm and 0.33, respectively. Each electrode was in the shape of a circle with a diameter of 43 mm. A sinusoidal voltage of about 8 kV peak-to-peak at a frequency of 12.5 kHz was applied using a high-voltage high-frequency supply (GBS Elektronik GmbH Minipuls 2.2) to the perforated electrode, and the plasma was generated along the rim of the perforated electrode. Since this technology is a gas-based sterilisation method, the sterilisation was performed in a chamber divided by volume into upper and lower compartments where the samples contaminated with bacteria and the plasma generator were located, respectively. The upper compartment had two valve ports used for purging and ventilation of the leftover ozone. Before use in the experiment, the temporal change in the ozone concentration inside the chamber after turning on the plasma device was measured by absorption spectroscopy of the 253.65 nm line of a mercury lamp.

### Test protocol

First, the appropriate ozone concentration and treatment time to decontaminate MDROs and *C. difficile* were deduced by conducting ozone sterilisation of bacterial cells placed on agar plates while varying ozone concentrations and treatment times. In the sterilisation, the chamber was first purged with ambient air, then the chamber was filled with ozone by turning on the plasma device. After treating samples with ozone for a given period of time, the leftover ozone was ventilated using a diaphragm pump. In the measurement, full 24 h culture samples (~ 10^8^ CFU/mL) were employed. Bacterial cell suspension samples (20 μL) were first serially diluted tenfold in sterile saline, then these samples were dispensed onto agar plates that were ozone-sterilised in the chamber. Thereafter, the duplicate samples consisting of an exposed and unexposed ozone sample were incubated at 37 °C for 24 h, and the number of colonies were counted for evaluation of sterilisation efficiency.

Next, with the sterilisation condition determined from the above study, the decontaminating effect of this technology for MDROs and *C. difficile* was evaluated using various material coupons (stainless steel, fabric, glass, plastic and wood coupons) frequently used in healthcare facilities. Full 24 h cultures (~ 10^8^ CFU/mL) were employed. Bacterial cell suspension samples (20 μL) were serially diluted tenfold in sterile saline, and then coupons were immersed in each of these diluted broths to assess contamination. The coupons taken out after immersing in the diluted broth were placed in sterile Petri dishes and dried at room temperature for 24 h. The Petri dish lids were placed over the coupons and were carefully placed in the test chamber. The Petri dish lids were removed, and the coupons were exposed to ozone gas of 500 ppm for 15 min. Control coupons were left covered in the biological safety cabinet and were not exposed to the ozone test conditions. Immediately after the ozone exposure, the coupons as well as those unexposed (i.e. controls) were mixed with a sterile saline solution using a vortex mixer to detach the bacteria from the surface. The eluted suspension was serially diluted tenfold in sterile saline, then the diluted bacteria were quantitatively plated onto blood agar plates for the aerobic bacteria or onto Brucella anaerobic blood agar plates for *C. difficile* and incubated at 37 °C for 24 h or anaerobically at 37 °C for 48 h in duplicate to determine the original inoculum concentration. Calculating the difference in the bacterial counts of the unexposed controls and of the exposed coupons yielded the logarithmic reduction in bacteria (i.e. sterilisation efficiency) under the test conditions.

### Atomic force microscopy (AFM) imaging of pathogens before and after ozone treatment and their sample preparation

Biological cells should be fixed on a flat plate for AFM imaging; thus, a flat and uniformly rough disc of mica, whose roughness scale was smaller than the size of the cells, was used as a base plate. The diameters and thicknesses of the discs were 20 mm and 0.21 mm, respectively. To firmly anchor the cells to the surface, the mica surface was coated with poly-L-lysine (200 μL) to make it positively charged as the cell membrane was negatively charged. After the poly-L-lysine coating, the mica discs were washed using 1 mL of de-ionised (DI) water three times and dried in air overnight. Bacterial cells were then loaded on the poly-L-lysine coated surface of mica by dispensing the diluted bacterial solution, resting for 30 min and then washing the mica surface using 1 mL of DI water.

Half of the samples were given the ozone treatment, and the morphologies of the mica plate surface loaded with *VRE*, *CRAB* and *C. difficile* spores were imaged via AFM (XE-7, park systems). An operation mode of the AFM was set to tapping mode, which has been the common method for taking images of biological cells. The microcantilever (OMCL-AC160TS, OLYMPUS Microscopy) that was designed for a non-contact mode was used in the experiment. The AFM image was recorded based on a probe scan rate of 0.5 Hz, resulting in an image resolution of 2048 × 2048 pixels.

## Results

### Experiments to find an effective sterilisation condition with a DBD plasma generator

To determine the conditions of the DBD plasma reactor for effective sterilisation, we ran serial experiments while altering ozone concentrations and exposure times using MDROs (VRE, CRE, CRPA and CRAB) and *C. difficile*. Figure 1b shows the temporal traces of the ozone concentrations for each test condition after the plasma device was turned on. The concentration increased logarithmically and was found to reach 300 ppm and 500 ppm after 1.5 min and 2.5 min, respectively. Preliminary tests on VRE had demonstrated that the minimum requirements for efficient decontamination of bacteria were an ozone dosage of 300 ppm for 10 min. Thus, in the following experiments, MDROs and *C. difficile* were exposed to two different ozone concentrations (300 and 500 ppm) and two different exposure times (10 min and 15 min). Sterilisation efficiencies were calculated for each setting of the ozone dosage and exposure time and they are tabulated in Table 1. Exposure to 300 or 500 ppm ozone for 10–15 min-exposure produced 2 or more log_10_ reduction in VRE overall. This high level of bacterial kill for CRE was achieved with 15 min of exposure at ozone concentrations of 300 or 500 ppm. High reduction in CRPA (> 7 log_10_) were achieved with exposure to 500 ppm of ozone for 15 min. At 300 ppm of ozone, the bacterial kill for CRAB was negligible; however, at 500 ppm ozone, there was a > 1.5 log_10_ reduction. Exposing *C. difficile* spores to 300 or 500 ppm ozone resulted in a > 2.5 log_10_ reduction.

**Table 1:** Effects of ozone on bacterial kill of VRE, CRE, CRPA, CRAB and *C. difficile* on agar plates.

		Ozone, ppm	Ozone treatment time, min	Log_10_ reduction ^a^
VRE	SCH-479	300	10	1.98
			15	7.98
		500	10	2.28
			15	7.98
	SCH-637	300	10	2.02
			15	2.32
		500	10	8.02
			15	8.02
CRE	SCH CRE-14	300	10	1.55
			15	8.15
		500	10	1.75
			15	2.45
	DK A-1	300	10	1.62
			15	8.10
		500	10	8.10
			15	8.10
CRPA	54	300	10	1.04
			15	1.69
		500	10	1.86
			15	7.56
	83	300	10	0.48
			15	0.86
		500	10	1.86
			15	7.56
CRAB	F-2487	300	10	0.02
			15	0.20
		500	10	1.40
			15	1.80
	SCH-511	300	10	0.26
			15	0.63
		500	10	1.39
			15	1.56
*C. difficile* spore		300	10	2.61
			15	2.63
		500	10	2.70
			15	2.73

*VRE* vancomycin-resistant *enterococci*, *MRPA* multidrug-resistant *Pseudomonas aeruginosa*, *MRAB* multidrug-resistant *Acinetobacter Baumanii*, *CRE* carbapenem-resistant *Enterobacterales* ^a^ numbers of colony-forming units per milliliter of test organisms.

### Antiseptic effect of gaseous ozone on various materials

**Figure 2:**
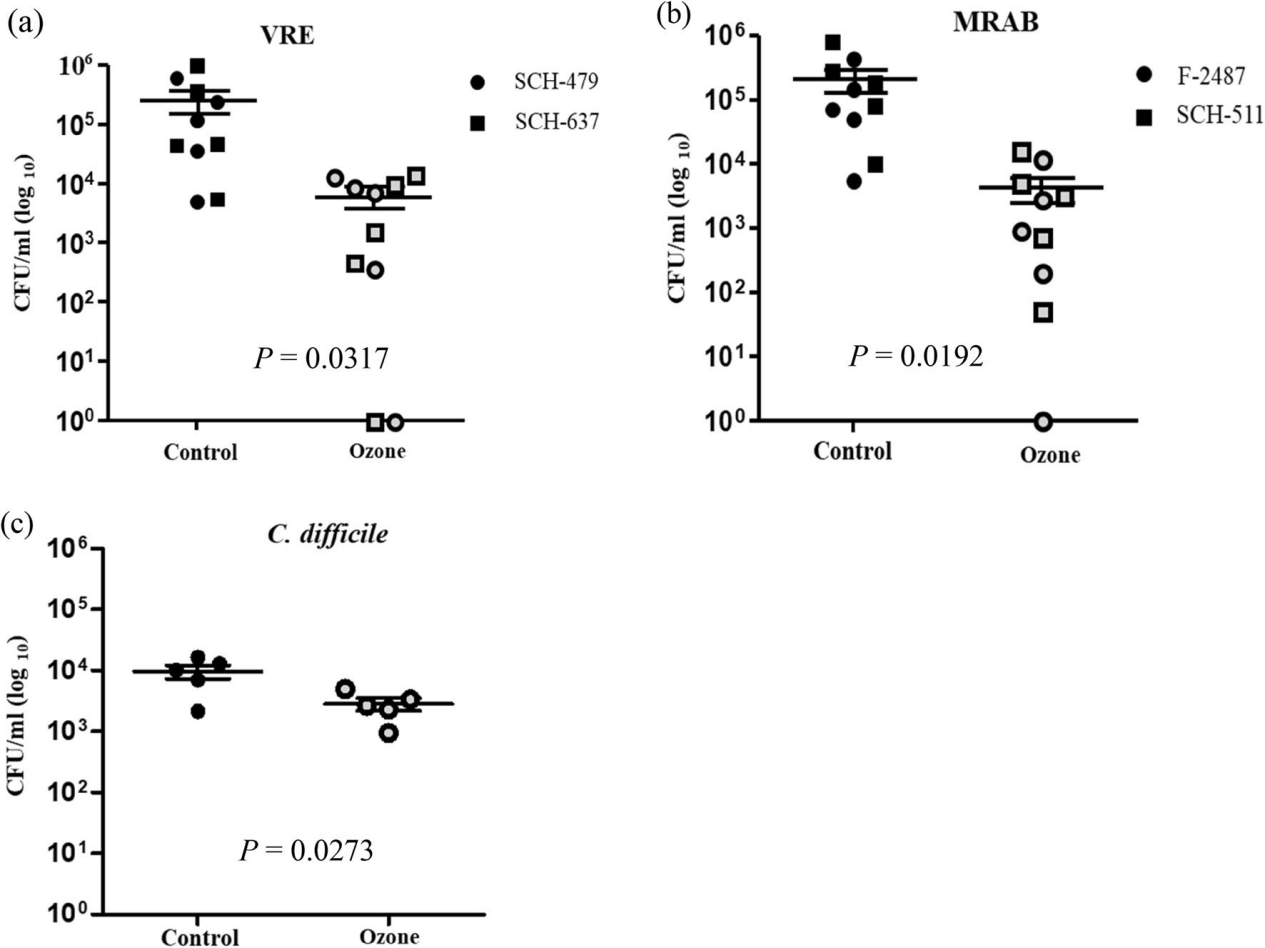
Scatterplot for effects of ozone on bacterial kill of (**a**) VRE, (**b**) CRAB and (**c**) *C. difficile* on various materials.

Based on the above experiments, sufficient requirements for inactivation of bacteria were concluded with an ozone dosage of 500 ppm for 15 min. VRE, CRAB and *C. difficile* spores were tested for the sterilisation effect of ozone on various materials, which were stainless steel, fabric, glass, plastic and wood, which are frequently used in a hospital environment. Their sterilisation efficiencies are tabulated in Table 2. Tested organisms were evaluated in duplicate. In VRE and CRAB, although about 2 or more log_10_ reductions were observed in stainless steel, fabric and wood surfaces, the antiseptic effect of ozone was inferior on glass and plastic surfaces. Spores of *C. difficile* were more resistant to ozone treatments than were all other organisms tested. To statistically examine the effects of ozone on the bacterial kill of VRE, CRAB and *C. difficile* with various materials, the differences between the control and treatment colony-forming units per millilitre counts on the different materials were compared using a *t*-test (Fig. 2). There were statistically meaningful differences in all strains, but more significant differences were observed with VRE and CRAB than with *C. difficile* spores.

**Table 2:** Effects of zone on bacterial kill of VRE, CRAB and *C. difficile* on various materials.

		Log_10_ reduction^a^				
		Stainless	Fabric	Glass	Plastic	Wood
VRE	SCH-479	2.02	1.94	1.46	0.98	3.69
	SCH-637	1.99	3.02	1.43	1.49	3.74
CRAB	F-2487	3.74	2.69	1.72	0.64	2.55
	SCH-511	2.30	2.41	1.07	1.76	2.05
*C. difficile* spore		1.13	0.30	0.20	0.80	0.49

*VRE* vancomycin-resistant *enterococci*, *MRAB* multidrug-resistant *Acinetobacter Baumanii,* ^a^numbers of colony-forming units per milliliter of test organisms.

### AFM imaging of VRE, CRAB and *C. difficile* spores before and after ozone treatments

**Figure 3:**
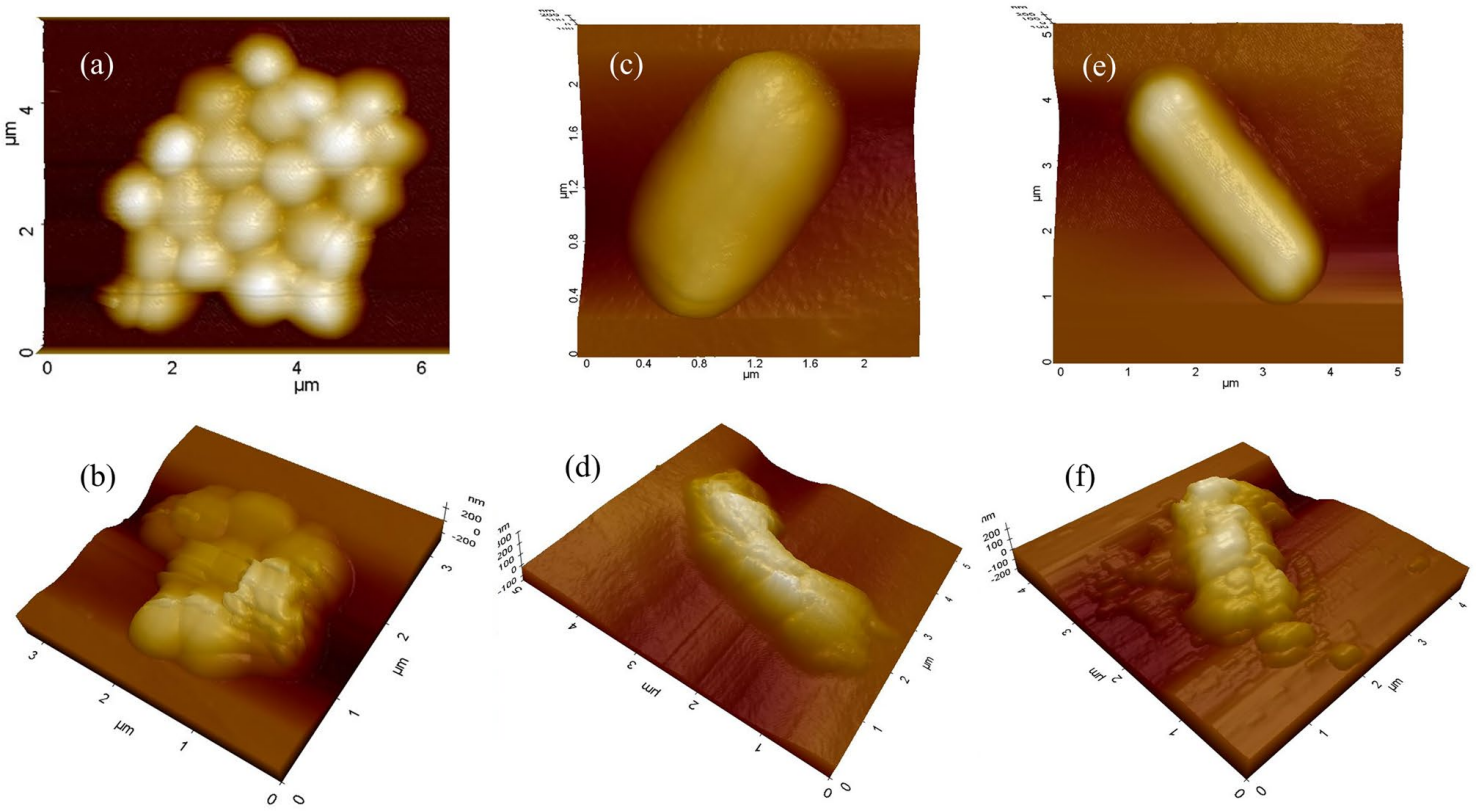
AFM images of VRE, MRAB and *C. difficile* spores (**a**, **c,** **e**) untreated and (**b**, **d**, **f**) treated with ozone at 500 ppm for 15 min. The images were plotted using the XEI program of Park Systems version 5.1.6 (XEI software, Suwon, Korea; https://www.parksystems.com/102-products/park-xe-bio).

AFM imaging is conducted on ozone-treated and untreated *VRE*, *CRAB* and *C. difficile* spores to investigate the sterilisation process by gaseous ozone in detail. Figure 3a,c and e show AFM images of untreated VRE, CRAB and *C. difficile* spore, respectively. The cells were smooth and intact, as shown by the 3D image. Figure 3b,d and f illustrate VRE, CRAB and *C. difficile* spores following exposure to the ozone treatment. For all test cells, not only did they shrink in size overall, but they also had a noticeably rougher surface after ozone exposure.

## Discussion

Our study showed that ozone produced by a DBD plasma device showed the ability of effective decontamination for MDROs and *C. difficile* spores, which known as the main cause of healthcare-associated infections. Also, in our study, considering that environmental contamination of MDROs and *C. difficile* spores could be a source of healthcare-associated infection, sterilisation effect of ozone was successful on materials that are mainly used in hospital facilities. After artificially contaminating materials, such as stainless steel, cloth, glass, plastic and wood with MDROs and *C. difficile* spores, a decontamination test was conducted with the DBD plasma device. As a result, although there was a difference in the decontamination effect depending on the materials, it showed ozone had significant ability for decontamination.

High-touch objects in hospital rooms warrant routine low-level disinfection. The standard decontamination of such objects is manual cleaning with a liquid disinfectant, such as a quaternary ammonium compound^13^. Even if strict adherence to the recommended disinfectant application is carried out, MDROs are difficult to remove by conventional environmental cleaning, which is typically by manual cleaning^14^. Therefore, new technologies, such as no-touch methods, are desirable. As a result, there has been an interest in gaseous disinfectants including hydrogen peroxide and ozone^10^. The advantage of gaseous disinfectants can reach places and objects inaccessible to conventional manual methods. Hydrogen peroxide has recently been used in healthcare settings; however, hydrogen peroxide is toxic in itself and should be dealt with according to strict handling procedures. Plasma sterilisation with hydrogen peroxide requires a relatively long purging time before the next sterilisation cycle. By comparison, ozone can be applied as a broad-spectrum antimicrobial that is effective against bacteria and viruses that can resist other disinfectants^8,11,15^. Moreover, ozone can be produced inexpensively with ambient air and does not require any additional toxic chemicals that may leave detrimental footprints on the environment, as it eventually decomposes into oxygen. Nevertheless, the reasons why ozone has not been widely used for disinfectant are as follows. Ozone is toxic to human health, thereby limiting its concentration below 0.07 ppm, averaged over an 8-h period^16^ so that ozone sterilisers have been developed and commercialised mainly for cleaning waste air. There is also the possibility of inhalation of the gas and an unpleasant odour being present after decontamination^5,8^. Ozone has not been actively used in healthcare settings yet. However, ozone can be used safely using sterilisation chambers and proper ventilation procedures, and the use of a catalytic converter can significantly speed up its removal. In this study, we demonstrated that a plasma ozone steriliser can be used for sterilisation of healthcare settings. We developed a device that had high sterilisation power, is easy to handle and had a rapid turnaround time for in-patient accommodations. In addition, we developed a sterilisation device with a simple structure that does not incur additional costs using ambient air. To date, there is no sufficient information on minimal ozone requirements for MDRO inactivation. The device used in our study had a simple configuration and short running-time, which is expected to be useful for sterilising equipment frequently.

Ozone’s mechanism of action for sterilisation has been not fully understood. Some studies have suggested that ozone destroys bacterial cell membranes, causing intracellular leakage and eventually cell lysis^17,18^. Ozone may disrupt cellular enzyme activity by reacting with thiol groups, and it may modify purine and pyrimidine bases in nucleic acids^19^. In this study, the morphologies of *VRE*, *CRAB* and *C. difficile* spores before and after ozone treatment were visualised and revealed not only the size had been shrunk but also the surfaces were significantly roughened, indicating damage or corrosion of the outermost membrane and inside materials due to the strong oxidising power of gaseous ozone. Such damage leads to cell inactivation depending on the severity of the cell alterations^18^.

*C. difficile* spores are known to be difficult to eliminate from hospital environments. The spores have long-term persistence in areas where they are shed^10,20^. Also, in this study, although the maximum log_10_ reduction in counts on agar plates was 2.73 when ozone was used at 500 ppm for 15 min, the sterilisation effect of ozone on various materials for *C. difficile* spore decreased. Therefore, different strategies could be considered to reduce *C. difficile* contamination in healthcare settings. It may also be useful to adjust the exposure time and intensity of ozone treatment with application only in *C.difficile* isolation rooms. And, we should remembered that ozone decontamination method cannot totally replace routine manual cleaning using disinfectant and antimicrobial polices also can be very effective for controlling *C. difficile*^5^. The effectiveness of ozone as a steriliser varied among different types of MDROs in this study. Effectiveness might depend on several factors, such as growth stage, the cell envelope and the efficiency of repair mechanisms^21,22^. The reasons for differences in the effectiveness of ozone sterilisation on the surface of each material may be relate to the formation of a biofilm. Previous studies have shown that *A. baumanni* and *E. faecium* confer increased environmental tolerance when existing as a biofilm^23–25^. Nevertheless, this study showed that ozone has a significant sterilisation effect on MDROs and *C. difficile* spores.

The limitation of our study is that we evaluated the ozone antiseptic effect after re-recultivation. It might result in overestimation of the number of survived bacterial cells.

Although this study was conducted to evaluate the effectiveness of ozone as a steriliser in a hospital environment, it is difficult to generalise our results to all hospital settings. Therefore, more studies are needed to examine the applicability and compatibility of this DBD ozone steriliser in actual hospital settings.

## Conclusion

The ozone generated by a DBD plasma reactor can provide a simple and valuable decontamination tool for MDROs and *C. difficile.* Ozone treatment may therefore be regarded as a valid alternate means of hospital environment disinfection.

## Data Availability

The datasets used and/or analyzed during the current study are available from the corresponding author on reasonable request.

## Abbreviations

MDRO: Multidrug-resistant organisms
DBD: Dielectric barrier discharge
VRE: Vancomycin-resistant *Enterococcus*
CRE: Carbapenem-resistant *Enterobacterales*
CRPA: Carbapenem-*resistant Pseudomonas aeruginosa*
CRAB: Carbapenem-resistant *Acinetobacter baumannii*
AFM: Atomic force microscopy
WHO: World Health Organization
ESBL: Extended-spectrum beta-lactamase
HCW: Healthcare worker
UV: Ultraviolet

## References

[1] WHO Global Strategy for Containment of Antimicrobial Resistance. https://www.who.int/drugresistance/WHO_Global_Strategy.htm/en/ Accessed.

[2] Dubberke ER, Olsen MA (2012). Burden of Clostridium difficile on the healthcare system. Clin. Infect. Dis..

[3] Boyce JM (2007). Environmental contamination makes an important contribution to hospital infection. J. Hosp. Infect..

[4] Kim YA, Lee H, K L. (2015). Contamination of the hospital environmental by pathogenic bacteria and infection control. Korean J. Nosocomial Infect Control..

[5] Dancer SJ (2014). Controlling hospital-acquired infection: focus on the role of the environment and new technologies for decontamination. Clin. Microbiol. Rev..

[6] Weber DJ, Rutala WA, Anderson DJ, Chen LF, Sickbert-Bennett EE, Boyce JM (2016). Effectiveness of ultraviolet devices and hydrogen peroxide systems for terminal room decontamination: Focus on clinical trials. Am. J. Infect Control..

[7] Siani H, Maillard JY (2015). Best practice in healthcare environment decontamination. Eur. J. Clin. Microbiol. Infect. Dis..

[8] Sharma M, Hudson JB (2008). Ozone gas is an effective and practical antibacterial agent. Am. J. Infect. Control..

[9] Seung-Lok Park J-DM, Lee S-H, Shin S-Y (2006). Effective ozone generation utilizing a meshed-plate electrode in a dielectric-barrier discharge type ozone generator. J. Electrostat..

[10] Moat J, Cargill J, Shone J, Upton M (2009). Application of a novel decontamination process using gaseous ozone. Can. J. Microbiol..

[11] Zoutman D, Shannon M, Mandel A (2011). Effectiveness of a novel ozone-based system for the rapid high-level disinfection of health care spaces and surfaces. Am. J. Infect Control..

[12] Wullt M, Odenholt I, Walder M (2003). Activity of three disinfectants and acidified nitrite against Clostridium difficile spores. Infect Control Hosp. Epidemiol..

[13] Ray A, Perez F, Beltramini AM, Jakubowycz M, Dimick P, Jacobs MR (2010). Use of vaporized hydrogen peroxide decontamination during an outbreak of multidrug-resistant Acinetobacter baumannii infection at a long-term acute care hospital. Infect Control Hosp. Epidemiol..

[14] Eckstein BC, Adams DA, Eckstein EC, Rao A, Sethi AK, Yadavalli GK (2007). Reduction of Clostridium Difficile and vancomycin-resistant Enterococcus contamination of environmental surfaces after an intervention to improve cleaning methods. BMC Infect Dis..

[15] Martinelli M, Giovannangeli F, Rotunno S, Trombetta CM, Montomoli E (2017). Water and air ozone treatment as an alternative sanitizing technology. J. Prev. Med. Hyg..

[16] Korean Ministiry of Environment. https://www.me.go.kr/mamo/web/index.do?menuId=586 (2022). Accessed 12 Jan 2022.

[17] Thanomsub B, Anupunpisit V, Chanphetch S, Watcharachaipong T, Poonkhum R, Srisukonth C (2002). Effects of ozone treatment on cell growth and ultrastructural changes in bacteria. J. Gen. Appl. Microbiol..

[18] Zhang YQ, Wu QP, Zhang JM, Yang XH (2011). Effects of ozone on membrane permeability and ultrastructure in Pseudomonas aeruginosa. J. Appl. Microbiol..

[19] Russell AD (2003). Similarities and differences in the responses of microorganisms to biocides. J. Antimicrob. Chemother..

[20] Whitaker J, Brown BS, Vidal S, Calcaterra M (2007). Designing a protocol that eliminates Clostridium difficile: A collaborative venture. Am. J. Infect Control..

[21] Broadwater WT, Hoehn RC, King PH (1973). Sensitivity of three selected bacterial species to ozone. Appl. Microbiol..

[22] Patil S, Valdramidis VP, Karatzas KA, Cullen PJ, Bourke P (2011). Assessing the microbial oxidative stress mechanism of ozone treatment through the responses of Escherichia coli mutants. J. Appl. Microbiol..

[23] Greene C, Wu J, Rickard AH, Xi C (2016). Evaluation of the ability of Acinetobacter baumannii to form biofilms on six different biomedical relevant surfaces. Lett. Appl. Microbiol..

[24] Flynn PB, Graham WG, Gilmore BF (2019). Acinetobacter baumannii biofilm biomass mediates tolerance to cold plasma. Lett. Appl. Microbiol..

[25] Ch'ng JH, Chong KKL, Lam LN, Wong JJ, Kline KA (2019). Biofilm-associated infection by enterococci. Nat. Rev. Microbiol..

